# Pallidal Hyperdopaminergic Innervation Underlying D2 Receptor-Dependent Behavioral Deficits in the Schizophrenia Animal Model Established by EGF

**DOI:** 10.1371/journal.pone.0025831

**Published:** 2011-10-12

**Authors:** Hidekazu Sotoyama, Yingjun Zheng, Yuriko Iwakura, Makoto Mizuno, Miho Aizawa, Ksenia Shcherbakova, Ran Wang, Hisaaki Namba, Hiroyuki Nawa

**Affiliations:** Department of Molecular Neurobiology, Brain Research Institute, Niigata University, Niigata, Japan; Tokyo Metropolitan Institute of Medical Science, Japan

## Abstract

Epidermal growth factor (EGF) is one of the ErbB receptor ligands implicated in schizophrenia neuropathology as well as in dopaminergic development. Based on the immune inflammatory hypothesis for schizophrenia, neonatal rats are exposed to this cytokine and later develop neurobehavioral abnormality such as prepulse inhibition (PPI) deficit. Here we found that the EGF-treated rats exhibited persistent increases in tyrosine hydroxylase levels and dopamine content in the globus pallidus. Furthermore, pallidal dopamine release was elevated in EGF-treated rats, but normalized by subchronic treatment with risperidone concomitant with amelioration of their PPI deficits. To evaluate pathophysiologic roles of the dopamine abnormality, we administered reserpine bilaterally to the globus pallidus to reduce the local dopamine pool. Reserpine infusion ameliorated PPI deficits of EGF-treated rats without apparent aversive effects on locomotor activity in these rats. We also administered dopamine D1-like and D2-like receptor antagonists (SCH23390 and raclopride) and a D2-like receptor agonist (quinpirole) to the globus pallidus and measured PPI and bar-hang latencies. Raclopride (0.5 and 2.0 µg/site) significantly elevated PPI levels of EGF-treated rats, but SCH23390 (0.5 and 2.0 µg/site) had no effect. The higher dose of raclopride induced catalepsy-like changes in control animals but not in EGF-treated rats. Conversely, local quinpirole administration to EGF-untreated control rats induced PPI deficits and anti-cataleptic behaviors, confirming the pathophysiologic role of the pallidal hyperdopaminergic state. These findings suggest that the pallidal dopaminergic innervation is vulnerable to circulating EGF at perinatal and/or neonatal stages and has strong impact on the D2-like receptor-dependent behavioral deficits relevant to schizophrenia.

## Introduction

Epidermal growth factor (EGF) and structurally related EGF-like peptides (such as neuregulin-1; NRG1) regulate GABAergic and dopaminergic development [1]–[5]. Genetic studies suggest that the genomic mutation or polymorphism for EGF, NRG1 and their receptors (ErbBs) is associated with schizophrenia risk [6]–[9]. Changes in the expression levels of EGF, NRG1 and ErbBs are also found in postmortem brains and peripheral blood of schizophrenia patients [10]–[13]. Both EGF and NRG1 are known to influence their own receptor binding and provoke signal cross-talks of other ErbB subtype(s) via ErbB1-4 receptor heterodimirization [14], [15]. Accordingly, abnormal ErbB signaling might be one of key features in schizophrenia neuropathology and/or etiology, although the pathophysiological nature of ErbB signaling in schizophrenia is largely unresolved [16], [17].

Among many environmental factors implicated in schizophrenia etiology, maternal viral infection and obstetric complications are suggested to play an important role in regulating vulnerability to schizophrenia [18]–[21]. These inflammatory insults often evoke abnormal cytokine signaling and perturb normal brain development [18], [21]. For instance, EGF levels in the amniotic fluid can sometimes reach the order of a microgram per liter, which is sufficient to occupy almost all EGF receptors (ErbB1) in the human fetus and lead to unfavorable uterine contractions and pre-term labor [22], [23]. To evaluate the impact of prenatal and perinatal ErbB hyper–signaling on neurobehavioral development, various ErbB ligands such as EGF and NRG1 were subcutaneously administered to rodent pups as their neurodevelopmental period matches the second trimester of human fetus having immature blood–brain barrier and initiating glial proliferation [24]. We found that ErbB ligands can penetrate the immature blood–brain barrier and reach brain neurons, resulting in various behavioral impairments at the post-pubertal stage [25]. For example, rats challenged with EGF as neonates display behavioral abnormalities in acoustic prepulse inhibition (PPI), latent inhibition of learning, social interaction, and methamphetamine sensitivity [26], [27]. The magnitude of the behavioral deficits, however, depends upon the type of ErbB ligands administered and the genetic background of the host animals [4], [27], [28]. In rodents, therefore, abnormal ErbB signaling in the prenatal and/or perinatal stage results in the neurobehavioral deficits [26]–[28] and/or dopaminergic abnormalities relevant to schizophrenia [4], although the neuropathologic mechanism underlying the individual deficits remains to be clarified.

Here, we prepared the animal model for schizophrenia by subcutaneously injecting EGF to newborn rats and studied the mechanism for their PPI abnormality. Based upon our latest finding on the neurotrophic interactions between EGF signaling and dopamine [5], [29], [30], we mainly characterized dopaminergic neuropathology of these rats using neurochemical and anatomical approaches. Furthermore we explored the pathologic mechanism underlying their behavioral deficits by pharmacologically manipulating local dopamine transmission.

## Results

### Upregulation of dopaminergic markers in the globus pallidus continuing until adulthood

**Figure 1:**
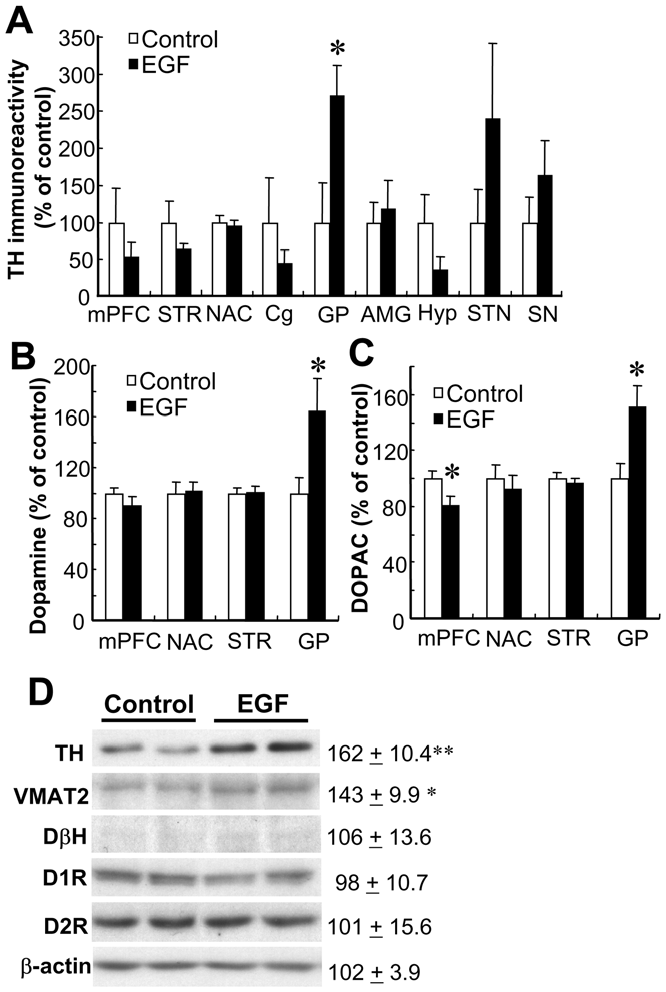
Effects of neonatal EGF challenge on tyrosine hydroxylase and dopamine metabolism at the adult stage. EGF or cytochrome c (control) was daily injected (s.c.) into neonatal rats from PND2 to PND10, and rats were bred until adulthood (PND 56). (A) Levels of tyrosine hydroxylase (TH) immunoreactivity in brain tissue homogenates were determined by ELISA and presented as a ratio of control levels (mean ± SEM). Tissue contents of dopamine (B) and its metabolite DOPAC (C) were measured by HPLC. (D) Tissue lysates from the globus pallidus of EGF rats and controls (8 weeks old, N = 4) were subjected to immunoblotting for antibodies directed against TH, vesicular monoamine transporter 2 (VMAT2), dopamine beta hydroxylase (DβH), dopamine D1 receptor (D1R), dopamine D2 receptor (D2R), and β-actin (an internal control). Immunoreactivity was measured by densitometric analysis and its percentage ratio to that of control rats was calculated (mean ± SEM). Abbreviations; medial prefrontal cortex (prelimbic cortex; mPFC), striatum (STR), nucleus accumbens (NAC), cingulate cortex (Cg), globus pallidus (GP), amygdala (AMG), hypothalamus (Hyp), subthalamic nucleus (STN), and substantia nigra (SN). *P<0.05, **P<0.01 by two-tailed t-test.

At the early postnatal stage of rats, EGF is verified to reach the brain through the immature blood-brain barrier and promote phenotypic development of midbrain dopaminergic neurons, leading to neurobehavioral abnormalities relevant to schizophrenia [26], [31]. However, there was a question of whether the neurotrophic influences on dopaminergic neurons can continue until the post-pubertal stage when rats develop the neurobehavioral abnormalities [26]. To address this question, here, we quantitated and compared protein levels of tyrosine hydroxylase (TH; a rate-limiting enzyme for dopamine synthesis) in various brain regions of EGF-treated and control rats at their adult stage using enzyme immunoassay (ELISA) [32], [33] (Fig. 1A). In the globus pallidus, TH protein levels were significantly higher in EGF-treated rats than in control rats (*P* = 0.035). There were no significant differences in the other brain regions in this ELISA (Fig. 1A). In the globus pallidus, there was concomitant elevation of tissue dopamine content (*P* = 0.030) and its metabolites, DOPAC (*P* = 0.014) (Fig. 1B, C) and HVA (*P* = 0.019, data not shown). We confirmed the pallidal hyperdopaminergic state by immunoblotting; protein levels of TH and VMAT2, but not those of DβH (a maker for noradrenergic neurons), were significantly elevated in EGF-treated rats at the adult stage (Fig. 1D).

**Figure 2:**
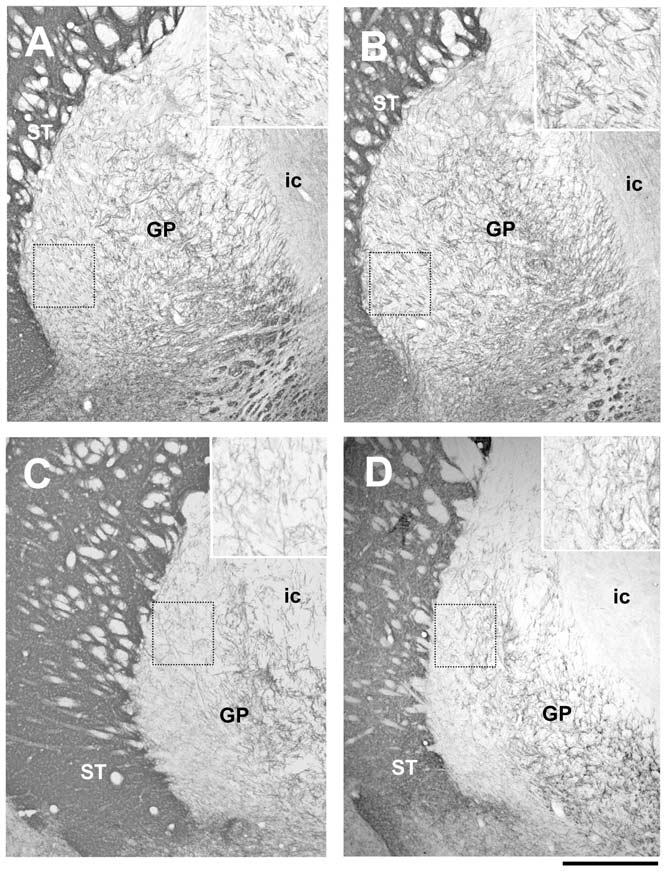
Immunohistochemical analysis of tyrosine hydroxylase-positive fibers and terminals in the globus pallidus. Coronal sections of the striatum containing medial and caudal globus pallidus (1.2 and 1.6 mm posterior from the bregma) were prepared from control and EGF-treated rats and immunostained with anti-TH antibody (N = 3–4 rats per group). TH-immunoreactive fibers in medial (A, B) and caudal (C, D) globus pallidus of control (A, C) and EGF rats (B, D) are shown. A lateral area of the globus pallidus is marked with a window, enlarged 2-fold, and presented in the top-right corner of each picture. ST; striatum, GP; globus pallidus, ic; internal capsule. Scale bar, 500 µm.

To determine whether the TH increase in the globus pallidus accompanies morphological alterations, we investigated the neuroanatomical features of dopaminergic fibers and terminals in EGF-treated rats (Fig. 2). In contrast to the neurochemical alterations, the anatomical difference in TH immunoreactivity was less marked. The density of TH-immunoreactive fibers was higher in EGF-treated rats only in the lateral area of medial and caudal globus pallidus neighboring the striatum. In rostral globus pallidus, however, we failed to detect the difference (data not shown).

### Antipsychotic effects on extracellular dopamine levels in the globus pallidus of EGF-treated rats

**Figure 3:**
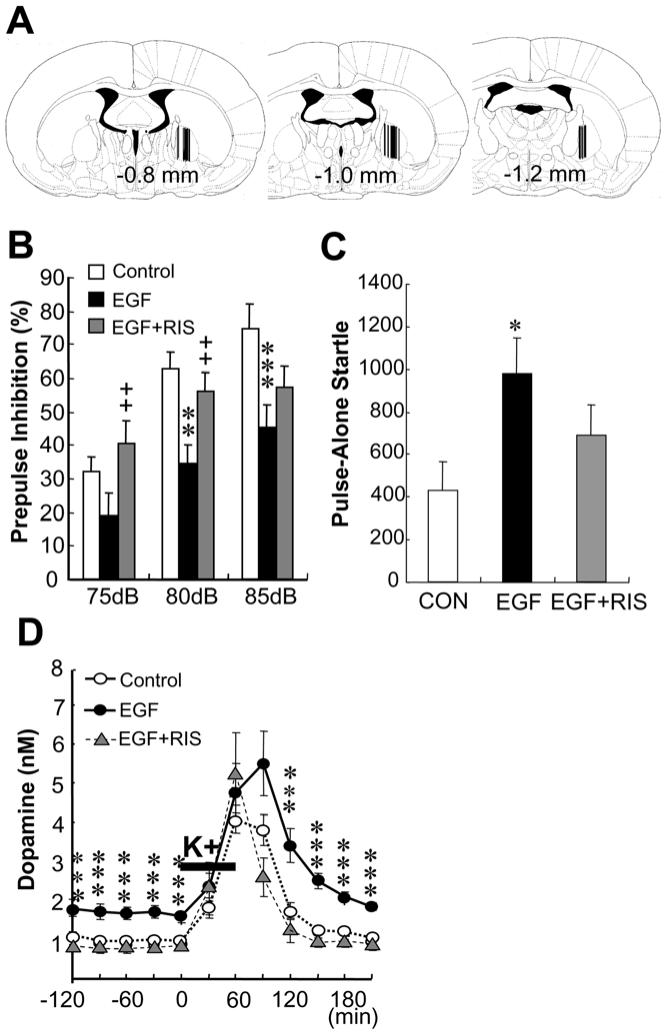
Pallidal dopamine release enhanced in EGF-treated rats and normalized with risperidone. Neonatal rats were treated with EGF or cytochrome c (control) as described in Figure 1. Risperidone (1 mg/kg, i.p.) was administered to some of the EGF-treated rats for 14 days at the adult stage. (A) The location of dialysis probe was examined and is shown in rat brain atlas. Six rats were excluded for incorrect probe placement. The digit represents the distance from the bregma. (B) PPI levels were monitored in control, EGF-treated and EGF+risperidone-treated animals following microdialysis. (C) Pulse-alone startle responses and PPI levels were monitored in control, EGF-treated and EGF+risperidone-treated animals following microdialysis. (D) Basal concentrations of dopamine in dialysates were monitored for 150 min, dopamine release was evoked by perfusion of 80 mM KCl over 60 min (solid bar), and then monitored over 150 min. Data represent dopamine concentrations in 30-min fractions (nM, mean ± SEM, N = 11–13 rats per group). There was a significant interaction between time and dopamine release [F(12,198) = 2.72, P = 0.002]. *P<0.05, ***P<0.001, compared with controls and ++P<0.01, compared with EGF-treated rats by Fisher's LSD.

Sensorimotor gating involves pallidal function and dopaminergic transmission [34]. We next monitored extracellular dopamine levels in the globus pallidus using a microdialysis technique and estimated a link between local dopamine concentration and sensorimotor gating (Fig. 3A). In agreement with the previous results [26], neonatal EGF treatment reduced PPI scores at the adult stage and an atypical antipsychotic agent ameliorated the PPI reduction (Fig. 3B). EGF-treated animals also exhibited an increase in pulse alone-startle amplitude compared with control animals but not with risperidone-treated animals [F(2,33) = 3.61, P = 0.038] (Fig. 3C). We found that basal extracellular dopamine levels (1.52±0.19 nM) in pallidal dialysates from EGF-treated rats were significantly elevated than in those from controls (0.85±0.06 nM; P<0.001, N = 11–13) but decreased by subchronic treatment with risperidone (0.67±0.08 nM; P<0.001, N = 11) [F(2,33) = 14.8, P<0.001] (Fig. 3D). Following potassium depolarization stimuli, extracellular dopamine levels were also elevated in EGF-treated rats but not in risperidone-administered EGF-treated rats [F(2,33) = 5.07, P = 0.008, N = 11–13]. The release difference at both the basal and evoked states suggests that EGF altered the capacity of net dopamine release but not the excitability of dopamine terminals. Of note, the overall changes in extracellular dopamine levels exhibited an opposite trend to that of the changes in PPI levels in the above experiments. These results raise the hypothesis that enhanced pallidal dopamine release might be associated with the PPI decrease in this model.

### Local reserpine administration to the globus pallidus ameliorates prepulse inhibition deficits

**Figure 4:**
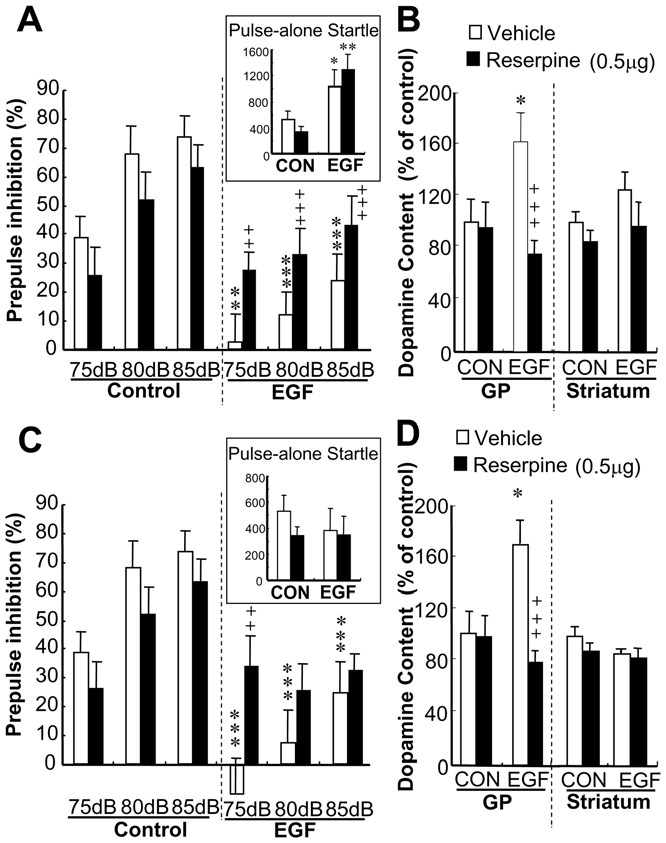
Effects of pallidal reserpine infusion on prepulse inhibition. (A) Startle responses of EGF and control (CON) rats were triggered with 120-dB tone pulse and PPI scores were measured with 75-, 80-, and 85-dB prepulse stimuli 120 min after local vehicle or reserpine infusion to both hemispheres of the globus pallidus. Pulse-alone startle responses to a 120-dB tone were measured in arbitrary units and are shown in the inset. (C) To match pulse-alone startle responses between EGF and control rats, 110-dB and 120-dB pulses were given to EGF and control rats, respectively, as shown in the insert. Following PPI test in (A) and (C), dopamine content was measured in the globus pallidus (GP) and neighboring striatum and shown in (B) and (D), respectively. Bars indicate mean ± SEM (N = 8–9 each). *P<0.05, **P<0.01, ***P<0.001, compared with vehicle-infused control rats; ++P<0.01, +++P<0.001, compared with vehicle-infused EGF rats by Fisher's LSD.

To confirm our hypothesis, we administered reserpine (an inhibitor of VMAT) or vehicle to reduce local dopamine content in synaptic vesicles and measured prepulse inhibition (Fig. 4A). A three-way ANOVA with EGF treatment and reserpine challenge as the between-subjects factors and prepulse intensity as the within-subjects factor revealed a significant interaction between EGF treatment and reserpine challenge [*F*_(1,29)_ = 7.81, *p* = 0.009]. Repeated Fisher's LSD revealed that reserpine challenge significantly improved PPI scores in EGF rats (*P* = 0.012). In contrast, reserpine did not alter PPI scores of control rats or pulse-alone startles of both groups of rats. Following the behavior tests, we monitored local dopamine contents to ascertain the regional specificity of the reserpine action (Fig. 4B). The dose of reserpine reduced the dopamine pool in the globus pallidus of EGF rats (P<0.001) but not in the neighboring striatum of EGF rats or in these regions of control rats.

There was a significant difference in pulse-alone startle amplitude between EGF-treated and control rats [*F*_(1,28)_ = 16.4, *P*<0.001 for EGF]. Our current design to evaluate reserpine action on %PPI might be inappropriate due to the basal difference in pulse-alone startle amplitudes of EGF and control rats [35]. We prepared another set of animals and gave the lower intensity of tone stimuli (110 dB for main pulses) to EGF-treated rats (Fig. 4C, D). The PPI difference between EGF and control groups as well as the risperidone effect still remained, even though there was no significant difference in the amplitudes of pulse-alone startles among groups.

**Figure 5:**
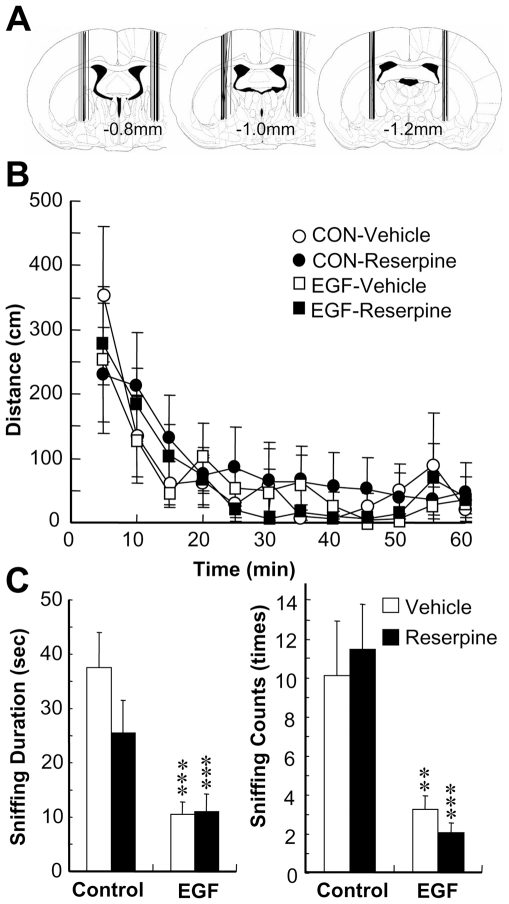
Influences of pallidal reserpine infusion on locomotor activity and social interaction. EGF and control (CON) rats received local reserpine- or vehicle-infusion to both hemispheres of the GP. (A) Cannula placement was confirmed in fixed brains, and two out of 36 rats were excluded from the final data analysis due to incorrect cannula placement. (B) Two hours after pallidal infusion, rats were placed in the automated activity monitoring chamber for 60 min. Data represent horizontal movement (cm) for every 5 min (mean ± SEM, N = 7–9 for each group). (C) Following locomotor test, an unfamiliar male rat was placed in the same chamber. The number and duration of sniffing behaviors of operated rats were counted for 10 min. Bars indicate mean ± SEM (N = 8–9 for each group). **p<0.01, ***p<0.001 by Fisher's LSD, compared with vehicle-infused controls.

To control the potential aversive effects of reserpine injection, we assessed the exploratory motor behaviors of reserpine-injected animals (Fig. 5A, B). We found no significant influence of reserpine on horizontal movement. As EGF-treated rats are known to exhibit a decrease in social interaction scores [26], we also evaluated the effects of reserpine on social behaviors following the above exploratory locomotor test (Fig. 5C). Two-way ANOVA for sniffing duration revealed a significant main effect of EGF [F(1,30) = 17.8, P<0.001] but no main effect of reserpine or no interaction between EGF and reserpine. The same statistical conclusion was drawn for sniffing counts as well. These results indicate that reserpine-induced pallidal dopamine reduction affected PPI levels of EGF rats, but did not influence their motor function or deficits in social behaviors.

### Effects of pallidal dopamine D2-like receptor blockade and activation on prepulse inhibition

**Figure 6:**
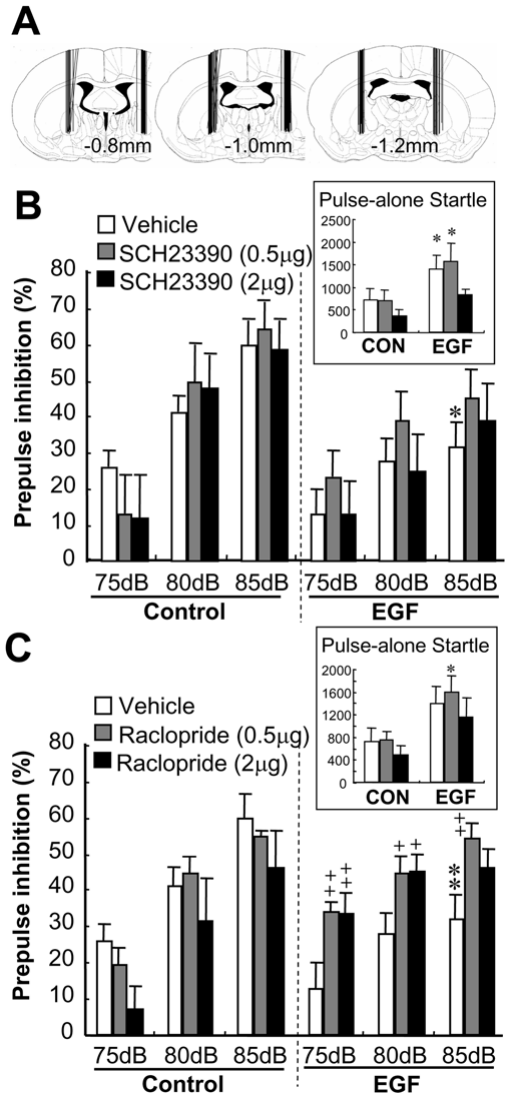
Effects of pallidal infusion of dopamine receptor antagonists on PPI deficits. Different doses of the D1-like receptor antagonist [SCH23390; 0 µg (vehicle), 0.5 µg or 2 µg per site] or the D2-like receptor antagonist [raclopride; 0 µg (vehicle alone), 0.5 µg or 2 µg per site] were administered to both hemispheres of the globus pallidus of EGF and control rats. (A) Cannula placement was confirmed in fixed brains, and four out of 69 rats were excluded from the final data analysis due to incorrect cannula placement. Rats receiving SCH23390 (B) or raclopride (C) were subjected to PPI test with 75-, 80- and 85-dB prepulse stimuli combined with a 120-dB startle tone. Pulse-alone startle responses to a 120-dB tone were measured in arbitrary units and are shown in the inset. Bars indicate mean ± SEM (N = 8–9 for each group). Data of rats receiving vehicle alone (control) were shared in (B) and (C). *P<0.05, **P<0.01, compared with vehicle-infused controls; +P<0.05, ++P<0.01, compared with vehicle-infused EGF rats by Fisher's LSD.

To test the possibility that the reserpine effects on PPI might result from its influences on the noradrenergic or serotonergic systems, we manipulated local dopaminergic transmission using dopamine receptor antagonists. We bilaterally administered a dopamine D1-like receptor antagonist, SCH23390 (0.5 µg and 2 µg per site), or a dopamine D2-like receptor antagonist, raclopride (0.5 µg and 2 µg per site), to the globus pallidus (Fig. 6A). SCH23390 failed to affect PPI scores at any dose in both groups of rats (Fig. 6B). In contrast, raclopride had differential effects in EGF and control rats [F(2,43) = 3.91 for EGF×raclopride dose, P = 0.028] (Fig. 6C). Post-hoc testing revealed that raclopride injection significantly ameliorated the PPI deficits of EGF rats (*P* = 0.019 and 0.043 for 0.5 µg and 2 µg raclopride, respectively) but not affected PPI levels of control rats. Similar to the results of the reserpine experiment, vehicle-treated control rats failed to react to the D2-like receptor antagonist at any dose.

**Figure 7:**
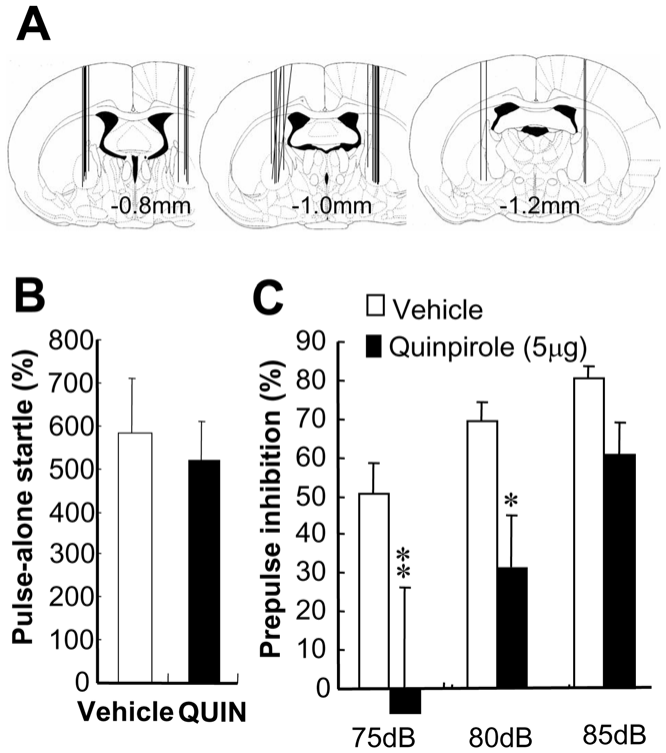
Effects of pallidal infusion of a dopamine D2-like agonist on PPI. A dopamine D2-like agonist, quinpirole (QUIN) (5 µg per site), or vehicle was administered to both hemispheres of the GP of naïve rats. (A) Cannula placement was confirmed in fixed brains, and one out of 18 rats was excluded from the final data analysis due to incorrect cannula placement. Fifteen minutes after quinpirole injection, pulse-alone startles (B) and PPI scores (C) were measured with 75-, 80- and 85-dB prepulse stimuli combined with a 120-dB startle tone. Bars indicate mean ± SEM (n = 8 or 9 each). *p<0.05, **p<0.01, compared with vehicle-infused controls by Fisher LSD.

To address whether a hyperdopaminergic state in the globus pallidus is sufficient to induce PPI deficits, we examined the effects of local stimulation of D2-like receptors. The bilateral challenge of naïve rats with quinpirole, a D2-like receptor agonist (5 µg per site), markedly disrupted PPI [F(1,16) = 6.50, P = 0.022] without altering pulse-alone startle (Fig. 7). These results support our argument that elevated dopamine release in the globus pallidus is responsible for the PPI deficits.

### Difference in cataleptic actions of a D2-like receptor antagonist on EGF-treated and control rats

**Figure 8:**
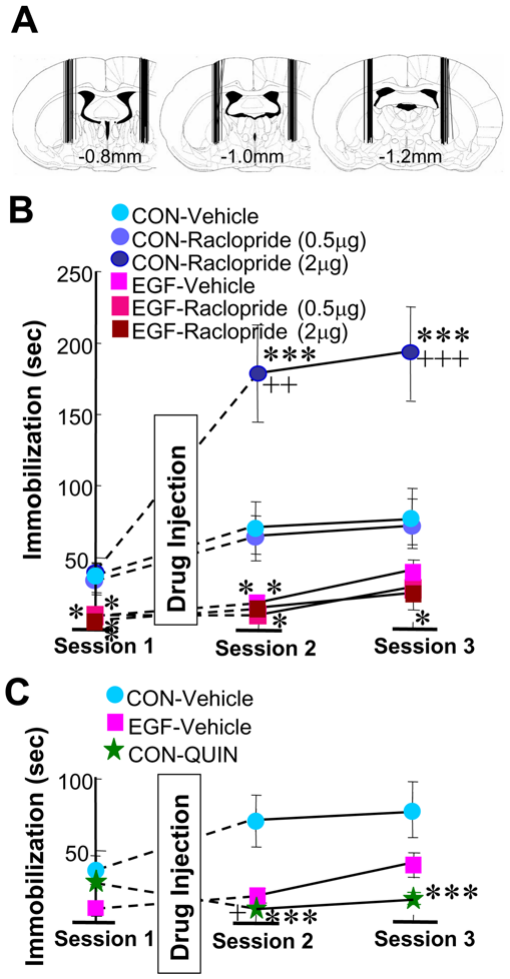
Measurement of immobility in the bar-hang test following pallidal infusion of the dopamine receptor antagonist and agonist. (A) Cannula placement was confirmed in fixed brains, and four out of 63 rats were excluded from the final data analysis due to incorrect cannula placement. The bar-hang test consisted of 3 blocks with 20-min intervals. Vehicle, raclopride (B; 0.5 and 2.0 µg per site) or quinpirole (QUIN) (C; 10.0 µg per site) was bilaterally injected into the globus pallidus of EGF and control (CON) rats after block #1. In each session we measured the latency until one of the paws of rats was removed from the horizontal bar (mean ± SEM, N = 8–9 rats each). *P<0.05, **P<0.01, ***P<0.001, compared with vehicle-infused control rats; +P<0.05, ++P<0.01, +++P<0.001, compared with the value of session #1 by Fisher's LSD. Note: The pallidal infusion of the D2-like receptor agonist to control rats (green star) significantly reduced the latency.

Because dopamine D2-like receptor blockade is known to induce cataleptic behaviors [36], we used the bar-hang immobility test to estimate the effects of the antagonist on catalepsy scores (Fig. 8A). Prior to drug administration, EGF-treated and control rats were subjected to the first session of the bar-hang test. There was a significant basal difference in bar-hang latency [F(1,43) = 19.0, P<0.001 for EGF] (Fig. 8B). Pallidal infusion of the D2-like receptor antagonist produced differential effects on EGF and control rats [F_(2,44)_ = 9.07, P<0.001 for raclopride dose×EGF]. The higher dose of raclopride significantly increased bar-hang latency in control rats (P<0.001) but not in EGF rats. Thus, EGF rats appear to be insensitive to the given doses of the D2-like receptor blocker in this test. These results rule out the possibility that the observed raclopride effects on PPI in EGF-treated rats might reflect its cataleptic action. Moreover, this experiment revealed the lower sensitivity of EGF-treated rats to pallidal D2-like receptor blockade.

Of note, the pallidal infusion of the D2-like receptor agonist quinpirole to naïve rats significantly diminished the bar-hang latency, compared with the latency before injection [F(2,24) = 4.54, P = 0.021] (Fig. 8C). The bar-hang latency of quinpirole-infused rats was significantly shorter than that of vehicle-infused controls (P<0.001) and indistinguishable from that of EGF-treated rats. All these results suggest that pallidal dopaminergic signals negatively regulate bar-hang latency. Therefore, the shorter bar-hung latency of EGF-treated rats supports our argument that pallidal dopamine function was up-regulated in these animals.

## Discussion

In the present investigation, we attempted to address the question of how EGF challenge at the perinatal stage alters later dopaminergic neurotransmission and dopamine-associated behavioral traits. The present analyses of this model provided us with the following information: 1) The hyperdopaminergic phenotype was maintained in the globus pallidus until adulthood. 2) The amounts of pallidal dopamine release were elevated at the adult stage of EGF-treated rats and normalized with antipsychotic treatment. 3) The reserpine-driven reduction in pallidal dopamine pool ameliorated the PPI deficits of EGF-treated rats. 4) The D2-like receptor blocker also normalized PPI levels of EGF-treated rats without affecting the catalepsy index. 5) Conversely, local administration of the D2-like receptor agonist to naïve rats caused deficits in PPI and bar-hang latency similar to that seen in EGF-treated rat. These results reveal that EGF exposure to rat pups produces persistent neurotrophic influences on the nigropallidal dopamine neurons and their functions. Thus, we postulate that the behavioral deficits of this EGF model, in part, can be ascribed to the D2 receptor-dependent hyperdopaminergic states of this basal ganglia circuit.

### Trophic actions and selectivity of epidermal growth factor peripherally administered

EGF and other ErbB ligands (such as neuregulin-1) are neurotrophic not only for midbrain dopaminergic neurons but also for GABAergic neurons and glial cells [1]–[5], [37], [38]. Although we have been investigating the phenotypic influences of peripheral EGF challenges on developing GABAergic and glial cells, these effects did not persist until the adult stage [2], [26], [31]. As such, the hyperdopaminergic influence found in the present investigation is the sole phenotypic change that we have detected in the adult stage of EGF-treated rats. Similar to this study, we find long-lasting influences of neonatal neuregulin-1 challenges on the dopamine system [4]. With the given wide-spread action of these ErbB ligands, however, we cannot rule out the possibility that undetected changes in GABAergic or glial phenotype or function still remain in this model [2], [5], [37], [38].

Individual dopaminergic systems of mesostriatal, mesolimbic, and mesofrontocortical pathways appear to be differentially regulated by individual neurotrophic factors [5], [37]–[39]. Among these neurons, a subset of dopaminergic neurons in the ventral tier of the substantia nigra pars compacta mainly express the EGF receptor (ErbB1), which constitute of highly branched dopaminergic neurons in the mesostriatal and pallidal pathways [5], [40], while the neuregulin-1 receptors (ErbB4) are most enriched in mesofrontocortical dopamine neurons [37]. Therefore, it is likely that mesostriatal dopaminergic population responded to exogenous EGF and contributed to the pallidal hyperdopaminergic states of the present animal model. In contrast to the neurochemical changes, the number of dopamine terminals in the globus pallidus was less remarkable. In this context, the neurotrophic feature of EGF on dopamine neurons remains to be fully characterized; its effects on cell survival, phenotypic enhancement, or terminal arborization. Our preliminary study failed to detect apparent difference in the number of dopamine neurons (data not shown).

In contrast to the present EGF model, the mice treated with neuregulin-1 as neonates, in which dopaminergic influences are most pronounced in the prelimbic cortex, exhibit less marked deficits in PPI but more severe impairments in social behaviors [4], [41]. The difference of the behavioral traits between the two models might reflect the distinct neurotrophic actions of EGF and neuregulin-1 on these cell subpopulations.

### Neuropathological implication of the globus pallidus for schizophrenia and its animal model

The globus pallidus is part of the indirect pathway of the basal ganglia circuit and receives dopaminergic innervation [42], [43]. The pathway initiates from the medium spiny neurons (MSN) in the striatum carry dopamine D2 receptors [44]–[46] and regulates sensorimotor gating, motor coordination, attention, learning and antipsychotic pharmacology [47]–[51]. Kodsi and Swerdlow (1995) showed that parts of globus pallidus, the ventral and caudal pallidum, are involved in sensorimotor gating [50]. The preceding report does not contradict the present finding although we did not distinguish these subregions of the globus pallidus. The dopaminergic abnormality in the indirect pathway represents one of the neuropathological features of this model and contributes to its behavioral deficits.

With respect to the antipsychotic effect of the D2 receptor antagonism in the indirect pathway, however, several controversies still remain; systemic administration of haloperidol failed to result in clear PPI improvement in EGF-treated rats [26]. In this context, neurobehavioral consequences of D2 receptor antagonism may significantly differ depending upon the brain regions where the antagonist acts [50]. Although we made the best efforts to minimize the technical artifacts and variations of cannula implantation, we cannot fully rule out aversive influences of the microsurgery on behavioral testing. The regional specificity of the drug actions should warrant future independent studies. As far as we compared the animals in control and experimental groups that equally received the surgery, however, the anti-dopaminergic manipulations produced the consistent results in behavioral tests.

The inhibition of dopamine D2 receptor at presynaptic sites of MSN fibers facilitates GABA release in the globus pallidus, leading to motor dysfunction such as catalepsy [42], [52]–[55]. In accordance with this cataleptic mechanism, our intrapallidal infusion of the D2-like receptor antagonist to control rats resulted in an increase in the catalepsy index while the D2-like receptor agonist conversely decreased the catalepsy index. We observed a similar D2-like receptor-dependency of the agonist and antagonist in the PPI paradigm. These results suggest the possibility that these behavioral deficits of EGF-treated rats involve the hyper-activation of D2-like receptors. However, the abnormalities of the present model in social behavior and pulse-alone startle appeared to be distinct from the pallidal dopamine neuropathology. In this context, EGF-treated rats should have the unrevealed structural or phenotypic alteration(s) underlying these behavioral impairments. Whether these impairments also involve the dopamine system remains to be investigated.

Of note, alterations in pallidal morphology and abnormal activation of this brain region are often implicated in schizophrenia. Brain imaging studies reveal pallidal structural abnormalities in schizophrenia patients [56]–[61]. Functional imaging also suggests an activation impairment in this brain region of patients [62], [63]. These abnormalities might have any association with the pallidal hyperdopaminergic states found in the present EGF model, especially if excess dopamine exerts neurotrophic and cell mitotic activities on pallidal cells and increases the tissue volume or function [64].

### Vulnerability of midbrain dopaminergic development to neurotrophic cytokines

Schizophrenia animal models established by maternal immune challenges or neonatal hypoxia appear to support the dopamine hypothesis for schizophrenia although several controversies still remain [65]–[67]. For example, the animal model established by maternal challenge with bacterial lipopolysaccharide shows a long-lasting increase in TH levels and dopamine turnover, at least, in the nucleus accumbens [68], [69]. Perinatal asphyxia and viral infection also result in distinct abnormalities in the dopamine system [70]–[74]. Our preceding study suggests that neuregulin-1, another ErbB receptor ligand, also induce several behavioral abnormalities relevant to schizophrenia, when neuregulin-1 is administered to newborn mice [4]. These findings indicate the possibility that the phenotypic and functional abnormalities in these immune/inflammatory models might involve EGF or other ErbB ligand(s) acting on midbrain dopamine neurons at prenatal or perinatal stages [25].

In conclusion, the developmental regulation of midbrain dopaminergic neurons is more vulnerable against peripheral cytokine signals than previously thought. The present results indicate that perinatal and potentially prenatal exposure to EGF or EGF-related cytokines may produce crucial and persistent impact on dopaminergic innervation and function in the indirect pathway of the basal ganglia circuit. In light of dopamine D2 receptor antagonism, which is commonly implicated in antipsychotic pharmacology, the present cytokine model may help to elucidate its antipsychotic mechanism as well as to validate the dopamine hypothesis for schizophrenia.

## Materials and Methods

### Ethics statement

All of the animal experiments described were approved by the Animal Use and Care Committee guidelines of Niigata University (Approval No18 on April 26, 2010) and performed in accordance with the guidelines of NIH-USA. Every effort was made to minimize the discomfort of the animals in addition to the number of animals used in the experiments.

### Animals

Male newborn Sprague-Dawley rats (SLC Ltd., Hamamatsu, Japan) were housed with a dam under a 12-h light/dark cycle (lights on 8:00 a.m.) in a plastic cage (276×445×205 mm). The rats were allowed free access to food and water. After weaning [postnatal day (PND) 20–30], rats were separated and housed with 2–3 rats per cage. Each adult animal (PND 56–94) was used in each experiment. Naïve Sprague-Dawley rats (all male, PND 56–70; SLC) were also used in control experiments. Recombinant human EGF (Higeta Shouyu Co., Chiba Japan) was dissolved in saline. EGF (0.875 µg/g) was administered subcutaneously (s.c.) each day to half of the littermates during PND 2–10 [26]. Control littermates received an injection of the same dose of cytochrome c (control protein) on the same schedule. The dose of EGF used in this study did not produce any apparent growth retardation in rats and mice [26], [28]. Some of adult rats daily received risperidone (1 mg/kg, i.p.; Janssen Pharmaceuticals Inc) or saline for 14 days.

### Enzyme-linked immunosorbent assay (ELISA)

Rats were anesthetized with halothane, and brains were removed and cut into 1-mm thick slices. Using published boundaries [75], we identified and punched out each brain region of interest. TH levels were measured using ELISA [10]. In brief, brain tissues were homogenized in 10 volumes of homogenization buffer [0.1 mM phenylmethanesulfonyl fluoride, 0.1 mM benzethonium chloride, 1 mM benzamidine (Sigma Chemical Co., St. Louis, MO), and 10 µg/ml aprotinin]. Brain homogenates were centrifuged at 14000× g for 20 min at 4°C, and the supernatants were stored at −80°C until use. Protein concentrations were determined using a Micro BCA kit (Pierce, Rockland, IL) with bovine serum albumin (BSA) as a standard.

Tissue homogenates or striatal lysates (standards) were loaded into ELISA plate wells coated with mouse monoclonal anti-TH antibody (a gift from Dr. Hatanaka and Dr. Takei). Plates were incubated with rabbit polyclonal anti-TH antibody (Chemicon, Temecula, CA) followed by incubation with anti-rabbit IgG β-galactosidase (1∶1000, American Qualex, San Clemente, CA). The fluorescence of the enzyme products from a reaction with 4-methylumbelliferyl-β-D-galactoside (MUG, Sigma) was measured using a microplate reader (COLONA electric Co., Ltd., Ibaraki, Japan).

### Immunoblotting

Each brain tissue was dissected as described above and homogenized in 200 µl lysis buffer [2% sodium dodecylsulfate (SDS), 10 mM Tris-HCl buffer (pH 7.4), 5 mM ethylenediamine-*N,N,N′,N′*-tetraacetic acid (EDTA), 10 mM NaF, 2 mM Na_3_VO_4_, 0.5 mM phenylarsine oxide], and boiled at 95°C for 5 min. After centrifugation at 12000 rpm for 20 min, the supernatant was harvested. The supernatant was mixed with 5× sample buffer [0.31 M Tris-HCl (pH 6.8), 10% SDS, 50% glycerol, 25% 2-mercaptoethanol] and boiled at 95°C for 5 min. Denatured protein samples were subjected to 7.5% SDS-polyacrylamide gel electrophoresis and transferred to a nitrocellulose membrane (Schleicher and Schull, Dassel, Germany) by electrophoresis. The membrane was probed with anti-TH (1∶2000, Chemicon), anti-vesicular monoamine transporter 2 (VMAT2) (1∶1000, Chemicon), anti-dopamine-beta-hydroxylase (DβH) (1∶500, Chemicon), anti-dopamine D1 receptor (1∶1000, Sigma) or anti-D2 receptor (1∶1000, Chemicon) antibodies. After washing, membrane immunoreactivity was detected using anti-rat, anti-rabbit, or anti-mouse immunoglobulin antibody conjugated to horseradish peroxidase (Jackson Immunoresearch Laboratory, West Grove, PA) followed by a chemiluminescence reaction (ECL kit, GE Health Science Inc., Tokyo, Japan) and exposure to X-ray films. Film images carrying a liner range of darkness of bands were subjected to film scanning and converted to the 8-bit digital data. Densitometric quantification of band intensity was performed with the free software Image J (National Institutes of Health, USA).

### Immunohistochemistry

Rats were anesthetized with halothane, perfused transcardially for 7 min with phosphate-buffered saline (150 mM NaCl, 0.1 M sodium phosphate; pH 7.5) followed by 4% paraformaldehyde in phosphate-buffered saline. Brains were removed and post-fixed in the same fixation solution for 24 h at 4°C. Fixed brains were immersed in 30% sucrose solution for 3–5 days, and frozen in resin (Tissue-Tek, Sakura Finetek U.S.A. Inc. Torrance, CA). Sections (40 µm) were cut with a cryostat (CM1510, Leica, Nussloch, Germany). After rinsing in Tris-buffered saline [TBS; 0.1 M Tris-HCl (pH 7.4), 150 mM NaCl] containing 0.2% Triton X-100, sections were pretreated with 6% BSA and 0.2% Triton X-100 in TBS and then incubated with anti-TH antibody (1∶1000, Chemicon) in TBS containing 3% BSA and 0.2% Triton X-100 for 48–72 h at 4°C. After rinsing in TBS/0.2% Triton X-100 three times, sections were incubated with biotinylated anti-rabbit immunoglobulin antibody (1∶200, Jackson Immunoresearch Laboratory). Immunoreactivity was visualized using a Vectastain Elite ABC kit (Vector Laboratories, Burlingame, CA) using diaminobenzidine as a substrate.

### Determination of monoamine contents

Each brain region was homogenized in 0.1 M perchloric acid containing 0.1 mM EDTA, and 100 nM isoproterenol. After centrifugation at 12000 rpm for 20 min, the supernatants and pellets were harvested. Concentrations of dopamine and its metabolites, 3,4-dihydroxyphenylacetic acid (DOPAC) and homovanillic acid (HVA), in supernatants were analyzed by HPLC-electrochemistry [4]. The mobile phase containing 50 mM trisodium citrate (pH 3.2), 25 mM NaH_2_PO_4_, 10 mM diethylamine, 0.03 mM EDTA, 2.5 mM 1-octane sulfonic acid sodium salt, 6% methanol, 1% dimethylacetamide was delivered at 0.5 mL/min. Monoamines were separated on an analytical HPLC column (CA-50DS, 4.6×150 mm, Eicom, Kyoto, Japan) and detected with a graphite electrode (WE-3G, Eicom) to which +700 mV was applied. Data analysis was performed with a data acquisition computer (Powerchrom, Eicom). Tissue pellets were homogenized in 0.5 N NaOH and subjected to protein determination with a Micro BCA kit (see above). Tissue monoamine contents were normalized with protein concentrations.

### Local drug administration to the globus pallidus

Control and EGF-treated rats (PND 56–70) were anesthetized with sodium pentobarbital (65 mg/kg, i.p.). After confirming deep anesthesia, a rat was mounted on a stereotaxic apparatus with an incisor bar set at 3.3 mm below the interaural line. The skull was exposed and two holes were drilled for bilateral implantation of guide cannulae (23 G stainless-steel pipes) into the GP. The stereotaxic coordinates were 0.9 mm anterior, ±3.0 mm lateral from the bregma, and 4.5 mm below the dura mater. After allowing the rat at least 10 days of recovery from surgery, a 30-G needle connected to Teflon tubing and a Hamilton syringe was placed 2 mm below the tip of the guide cannula. The drug (0.5 µl) was injected over a period of 30 sec, and the needle left in place for an additional 30 sec. Rats were placed in their home cage for 5–15 min, to allow for local diffusion of the drug; the rats were then subjected to behavioral tests (see below). When rats received reserpine, rats were placed in their home cage for 120 min to deplete the local dopamine pool. The cannula position was confirmed after the completion of the behavioral tests (see below).

Reserpine (Daiichi Sankyo Pharmaceutical Inc., Tokyo, Japan) was dissolved in phosphate-buffered solution (pH 4.0) containing 3 mg/mL DL-methionine and 70 mg/mL propylene glycol. Conventional dopamine receptor ligands, SCH23390, raclopride and quinpirole, were all obtained from Sigma and dissolved in 10% dimethyl sulfoxide (DMSO) in saline (vehicle).

### Microdialysis

Rats were anesthetized with sodium pentobarbital (65 mg/kg i.p.) and mounted in a stereotaxic apparatus. The skull was exposed and a hole was drilled for unilateral implantation of a guide cannula (AG-8, Eicom) into the GP. The stereotaxic coordinates were 0.9 mm anterior; 3.0 mm lateral from the bregma, and 4.8 mm below the dura mater. After allowing the rat at least 10 days of recovery from surgery microdialysis experiments were performed.

The microdialysis probe (2 mm active area, A-I-8-02, Eicom) was connected to Teflon tubing (0.65 mm o.d., 0.12 mm i.d.; Bioanalytical Systems Inc., West Lafayette, IN). The rat was perfused with artificial cerebrospinal fluid (pH 7.0) containing 147 mM NaCl, 2.7 mM KCl, 1.2 mM CaCl_2_, and 0.5 mM MgCl_2_ at a flow rate of 0.7 µL/min. Dialysate was discarded to obtain a steady state for at least 18 h after implantation of the probe, and then dialysate samples were collected every 30 min. The first five fractions were collected to determine basal levels of dopamine. The perfusion medium was switched to the medium containing a high concentration of potassium (80 mM KCl, 69.7 mM NaCl, 1.2 mM CaCl_2_, 0.5 mM MgCl_2_; pH 7) for 60 min (for two fractions). The perfusion medium was then switched back to the original medium and five fractions were additionally collected. In all, total 12 fractions were collected.

Dopamine in the dialysates was determined by HPLC with electrochemical detection. The mobile phase containing 48 mM citric acid, 24 mM sodium acetate, 10 mM NaCl, 0.5 mM EDTA, 2.5 mM SDS, and 16% acetonitrile (pH 4.8) was delivered at 50 µL/min. Dopamine was separated on an analytical column (BDS Hypersil C18 1×100 mm, Thermo Fisher Scientific, Yokohama, Japan) and detected with a 3 mm glassy carbon electrode detector (Unijet flow cell; Bioanalytical Systems Inc.) to which +550 mV was applied. Data analysis was performed with the analysis software (Epsilon LC; Bioanalytical Systems Inc.). Data were not compensated with the recovery rate.

### Confirmation of cannula positioning

After local drug injection or microdialysis, rats were deeply anesthetized with halothane and decapitated. Brains were quickly removed and fixed in 4% paraformaldehyde for 3 days. Fixed brains were cut into 50-µm sections using a vibratome (Dosaka EM Ltd., Kyoto, Japan). Each section was stained with 0.5% cresyl violet solution. The location of a microdialysis probe or injection needle was determined under a microscope according to a stereotaxic atlas [75]. Animals that exhibited incorrect cannula placement were removed from the data analysis.

### Measurement of acoustic startle and prepulse inhibition of startle response

Acoustic startle amplitude was measured in a startle chamber (SR-Lab Systems, San Diego Instruments, San Diego, CA) [34]. Rats were placed into a startle chamber with 70-dB background noise. Five minutes later, the startle amplitude was recorded in a session that included multiple trial types: (i) a 120-dB 40-ms noise burst presented alone (pulse); (ii–iv) 40-ms 120-dB noise burst following prepulses by 100 ms (20-ms noise burst) that were 5, 10, and 15 dB above background noise (i.e., 75-, 80-, 85-dB prepulse, respectively); and (v) no stimulus (background noise alone). The percentage PPI of startle responses was calculated as: 100−[(startle response on prepulse-pulse stimulus trials – no stimulus trials)/(pulse-alone trials – no stimulus trials)]×100. To match the magnitude of pulse alone startles between groups, a 120-dB noise burst was replaced with a 110-dB noise burst in some experiments.

### Analysis of locomotor activity

Side effects of reserpine were estimated by monitoring spontaneous locomotor activity under novel conditions. Reserpine-infused rats were placed in an open field box (45 cm length×45 cm width×30 cm height, MED Associates, St. Albans, VA, USA) under a moderate light level (200 Lx). Line crossings and rearing counts were measured by photo-beam sensors (25 mm intervals for horizontal axis and 150 mm for vertical axis).

### Social interaction test

The index for social interaction of rats was measured according to Futamura et al. (2003) [26]. Following the above locomotor test, reserpine- or vehicle-infused rats were exposed to an unfamiliar male rat that was housed in another cage, and was age, body-weight, and gender-matched. All tests were videotaped and scored in blind. Scoring of social interaction times and duration was based on sniffing behaviors, defined as active chasing of the partner, shaking the nose near the partner, and contacting the partner with the nose.

### Measurement of immobilization levels in the bar-hang test

Immobilization levels and the cataleptic effects of drugs were measured using a bar-hang test method [76]. In the bar-hang test, the front paws of the rat were gently placed on a horizontal metal bar (5 mm diameter) and placed 10 cm above ground level. The test was terminated when the paw of animal was released from the bar or 300 sec had passed, and the total time until the animals removed the paw from the bar was recorded. Rats were subjected to three blocks (separated by 20-min intervals) of three trials. Scores at the three different time blocks (after 1-h acclimation, 20 and 40 min after drug infusion) were monitored for comparison purposes.

### Statistical analysis

All data are expressed as means ± SEM. Statistical differences in the behavioral data were determined by analysis of variance (ANOVA). When univariate data were obtained from two groups, two-tailed *t*-test was used for comparison. Behavioral scores were initially subjected to factorial ANOVA using neonatal EGF treatment (two levels) and local drug infusion (two or three levels) as between-subjects factors, and prepulse magnitude (three levels) or test session (two levels) as a within-subjects factor. As the initial analyses yielded a significant factorial interaction, data were subjected to a Fisher's LSD *post-hoc* test with or without repeated measure. The interaction of a within-subjects factor with a between-subjects factor was estimated by multivariate analysis of variance (MANOVA). Individual statistical differences between data points are shown in the figures. Correlations between dopamine release and PPI were examined by Pearson's correlation analysis. A *p* value less than 0.05 was regarded as statistically significant. Statistical analyses were performed using StatView software (SAS Institute Inc., Cary, NC, USA). *N* values in parentheses represent the number of animals used in each group.
